# Structural insight into 6-OH-FAD–dependent activation of hFSP1 for ferroptosis suppression

**DOI:** 10.1038/s41421-024-00723-7

**Published:** 2024-08-20

**Authors:** Hongying Lan, Yu Gao, Ting Hong, Zihan Chang, Zhengyang Zhao, Yanfeng Wang, Feng Wang

**Affiliations:** Key Laboratory of Molecular Medicine and Biotherapy in the Ministry of Industry and Information Technology, Department of Biology, School of Life Sciences, Beijing Institute of Technology, Beijing, China

**Keywords:** X-ray crystallography, Protein folding

Dear Editor,

Ferroptosis is characterized by unchecked lipid peroxidation and disruption of the cell membrane, resulting in mortality^1^. Human ferroptosis suppressor protein 1 (hFSP1), which utilizes 6-OH-FAD as the primary cofactor, prevents lipid peroxidation by oxidizing NAD(P)H and reduces coenzyme Q/vitamin K on the membrane to suppress ferroptosis^2–5^. In addition, 6-OH-FAD but not FAD was observed to bind hFSP1 intrinsically in both *E. coli* and Sf9 expression systems. More recently, the crystal structures of hFSP1-6-OH-FAD-NADP^+^ and hFSP1-6-OH-FAD-NADH have been reported^6,7^. Given the implications of blocking ferroptosis, interfering with hFSP1 chemically has emerged as a promising clinical strategy for resisting tumour growth associated with ferroptosis^3,8,9^.

Nevertheless, the structure representing the initial state of hFSP1 without any bound substrate and the underlying mechanism behind the preferential selection of hFSP1 for 6-OH-FAD remain elusive. This hinders a comprehensive understanding of the entire NADH/NADPH oxidation process in hFSP1.

To investigate the role of 6-OH-FAD in hFSP1 activation, we first attempted to determine the crystal structures of full-length hFSP1, which unfortunately suffered from poor expression. Reasoning that this problem might be caused by the flexible surface loops in the N-terminus and C-terminus, we generated many truncated hFSP1 variants with varying lengths of N-terminal or C-terminal deletions. After numerous trials, well-diffracted crystals were only successfully obtained from a recombinant hFSP1 variant with the first nine amino acid residues deleted, which has comparable activity to that of full-length hFSP1. The intrinsic cofactor extracted from recombinant hFSP1 exhibited a main peak at an *m*/*z* value of 800.276, indicating that hFSP1 primarily utilizes 6-OH-FAD as its cofactor (Fig. 1b). Next, we determined the crystal structures of hFSP1-6-OH-FAD, hFSP1-6-OH-FAD-NAD^+^, and hFSP1-6-OH-FAD-NADP^+^ (Supplementary Table S1). As previously described^6,7^, hFSP1 shows the typical characteristics of the type II NADH: quinone oxidoreductase (NDH-2) family and is divided into three domains: the 6-OH-FAD-binding domain (residues 10–111 and 246–327), the NAD(P)H-binding domain (residues 112–245), and the C-terminal membrane-anchoring domain (CTD, residues 328–373) (Supplementary Fig. S1a–c). The 6-OH group is reflected by an extra unambiguous electron density at the carbon 6 position of the isoalloxazine ring of FAD in all three structures (Fig. 1a), further confirming that 6-OH-FAD is the cofactor intrinsically associated with our recombinant hFSP1. Similarly, the electron densities for both NAD^+^ and NADP^+^ within hFSP1 are clearly defined in the crystal structures of hFSP1 complexed with NAD(P)^+^ (Fig. 1a). A search of the Dali database revealed that hFSP1 shares considerable structural similarities with cFSP1 (Protein Data Bank (PDB): 7XBI), Ndi1 (PDB: 4G6G), hAIF (PDB: 1M6I), and c.thNDH2 (PDB: 4NWZ).

**Fig. 1:**
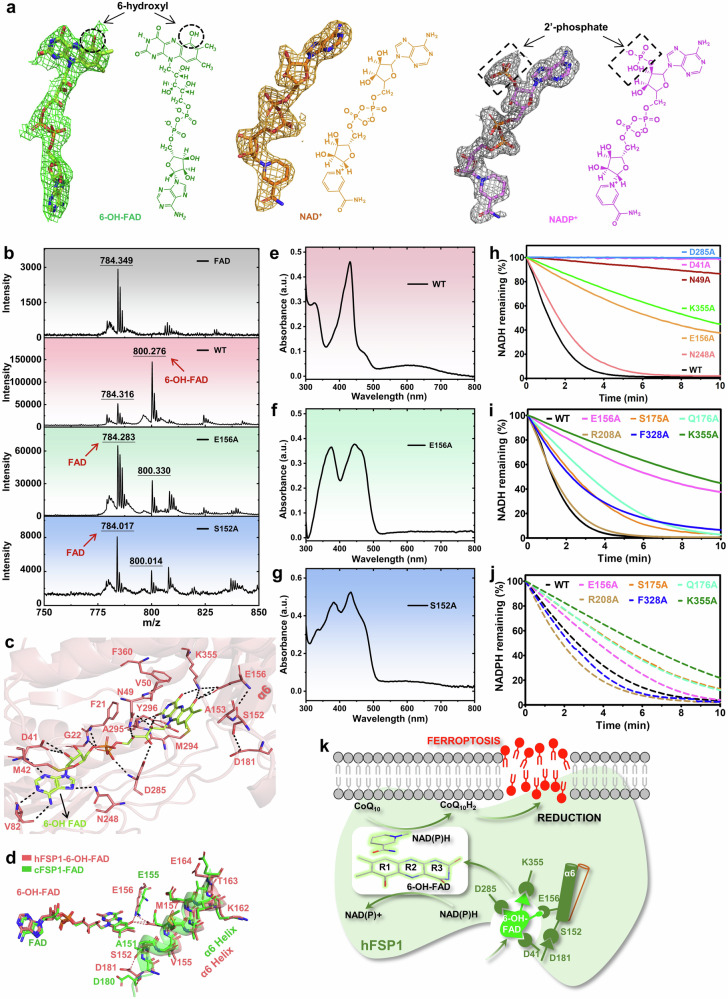
Characterization of hFSP1 bound to 6-OH-FAD and NAD(P)^+^. **a** 2*F*o–*F*c electron density map contoured at the 1.0 σ level of 6-OH-FAD (left, green mesh), NAD^+^ (middle, brown‒yellow mesh), and NADP^+^ (right, grey mesh) in hFSP1. The chemical structure corresponding to the factor combined with hFSP1 is on the right side of its electron density map. **b** Representative MALDI-TOF MS peaks for commercial FAD (FAD), the cofactors bound to wild-type hFSP1 (WT), hFSP1-E156A (E156A), and hFSP1-S152A (S152A). The arrows indicate the molecular weights corresponding to the cofactors. **c** 6-OH-FAD-binding pocket. 6-OH-FAD is shown in lemon green. The residues of hFSP1 that are involved in the interaction are shown in pink. The black dashed lines represent hydrogen bond interactions. **d** Structural superposition of hFSP1-6-OH-FAD (in pink) with cFSP1-FAD (PDB: 7XPI, in green); the cofactors and several key residues involved in 6-OH-FAD binding are highlighted. **e**–**g** Results of the ultraviolet-visible spectroscopy assay for wild-type hFSP1 and its mutants: **e** wild-type hFSP1 (WT), **f** E156A, and **g** S152A. **h** Representative reaction curves of hFSP1 and its mutants (D41A, N49A, E156A, N248A, D285A, and K355A) in an enzyme activity assay for measuring NADH consumption at 340 nm. **i**, **j** Assay for NAD(P)H consumption at 340 nm in wild-type hFSP1 (WT, black trace) and its variants E156A (pink trace), S175A (bright yellow trace), Q176A (teal trace), R208A (golden brown trace), F328A (blue trace), and K355A (Spanish green trace). **i** NADH consumption is depicted with a solid line, while **j** NADPH consumption is represented by a dashed line. **k** Schematic diagram of 6-OH-FAD-dependent ferroptosis suppression by hFSP1.

In our structures, the additional hydroxyl group at the 6th position of the isoalloxazine ring in 6-OH-FAD generates a new interaction with hFSP1, which is absent in other NDH-2 family members (Fig. 1c and Supplementary Fig. S2a–e). The 6-OH group is engaged by several residues of hFSP1, mainly Ser152, Glu156, and Lys355. The hydroxyl group of Ser152, an initial residue within the α6 helix, hydrogen bonds with the carboxyl group of Asp181 to facilitate repositioning of the α6 helix (Fig. 1c). Consequently, Glu156 on the α6 helix, which is a highly conserved residue among the NDH-2 family members and was identified as crucial for hFSP1 activity in previous research^2,10,11^, contacts the main-chain carbonyl group of Ser152 via a typical hydrogen bond (Fig. 1c). This interaction directs residue Glu156 to bind 6-OH-FAD. Moreover, structural comparison between hFSP1 and cFSP1 revealed conformational differences in other regions of the α6 helix, including Val155, Met157, Lys162, Thr163, and Glu164 (Fig. 1d). These residues likely indirectly promote the binding of Glu156 to the 6-OH group through repositioning and stabilization of the α6 helix. Additionally, Glu156 hydrogen bonds to Lys355, enabling the side chain of Lys355 to be located within the hydrogen bonding distance of the isoalloxazine ring of 6-OH-FAD (Fig. 1c). In summary, hFSP1 orchestrates a spatial rearrangement, driven by the interplay between Ser152 and Asp181, to enhance its selective binding to 6-OH-FAD.

Moreover, the isoalloxazine ring of 6-OH-FAD is nestled on the hydrophobic pocket formed by the residues Val50, Ala153, Met294, and Phe360 and hydrogen bonds with the main-chain amides of Ala295 and Tyr296. The adenine ring of 6-OH-FAD is stabilized by the carbonyl group of Val82 and the side chain of Asn248, while the 2-OH group in the pentose moiety interacts with Asp41 through hydrogen bonding. In addition, the pyrophosphate moiety interacts with the amino groups from Phe21, Gly22, and Asp285, and the ribitol section contacts the side chains of Asp285 and Asn49 (Fig. 1c).

To confirm the crucial residues responsible for 6-OH-FAD binding, multiple hFSP1 mutants, including E156A, D41A, N49A, N248A, D285A, and K355A, were generated to assess their impacts on 6-OH-FAD binding and enzymatic activity. Notably, the E156A mutant appears drastically bright yellow, in addition to the green color observed during purification of the wild-type hFSP1 (Supplementary Fig. S3), and its enzyme activity also markedly decreased (Fig. 1h). This color change may be caused by the exchange of the cofactor bound to hFSP1, since the color of FAD is yellow, whereas that of 6-OH-FAD is green. Next, we identified the cofactor extracted from the E156A mutant via MALDI-TOF MS. As expected, a main peak occurred at an *m*/*z* value of 784.283, which is in accordance with the theoretical molecular weight of FAD (Fig. 1b). The faint peak at *m*/*z* 800.330, which corresponds to 6-OH-FAD, possibly originates from its nonspecific weak binding to E156A, since 6-OH-FAD differs from FAD only in the extra hydroxyl group. Moreover, we utilized ultraviolet-visible spectroscopy to further verify the cofactors in both the wild-type and E156A mutant strains. Unlike the characteristic peaks for 6-OH-FAD in wild-type hFSP1 (with a peak at 430 nm and a broad peak at approximately 600 nm), the characteristic peaks of FAD at approximately 378 nm and 459 nm are detected in the E156A mutant (Fig. 1e, f)^5^. These observations support the importance of Glu156 in the binding of hFSP1 to 6-OH-FAD, which might be correlated with the cellular resistance to ferroptosis driven by hFSP1, as described in prior reports^2,10,11^. In addition, we also observed that both the D41A and D285A mutants are completely unable to bind 6-OH-FAD or FAD (Supplementary Fig. S4a, b), which might explain the findings of our experiments and a recent report that these two mutations nearly abrogate the oxidoreductase activity of hFSP1 in vitro (Fig. 1h)^10^. Furthermore, the N49A and K355A mutants also weakened the enzymatic function of hFSP1 to some extent, whereas the N248A mutation had little influence on its enzymatic activity, which is in line with their binding affinity for 6-OH-FAD (Fig. 1h and Supplementary Fig. S4c–e). Overall, our observations suggest that Asp41, Asn49, Asn248 and Asp285 are likely required for the binding of hFSP1 to the 6-OH-FAD body, whereas Glu156 and Lys355 are associated with its binding to the 6-OH group.

To elucidate the molecular determinants of preferential 6-OH-FAD selection in hFSP1, we conducted comprehensive structural dissection and multiple sequence alignment of the amino acids surrounding Glu156. Interestingly, the corresponding residues of Ser152 and Lys355 in hFSP1 orthologues from lower organisms to fungi, insects and even poultry appear to be hydrophobic amino acids in most cases (Ala, Pro, or Phe at position 152; Pro, Leu, Glu, or Arg at position 355) (Supplementary Fig. S5). In amphibian and reptile species, Ser is predominantly observed at the 152nd position, with nonpolar, uncharged Ala occasionally occurring. However, Ser occupies this position in nearly all mammals, including chimpanzees, antelopes, and platypuses. As expected, the Ala151 residue in cFSP1 is unable to form a hydrogen bond with the invariant residue Asp180 (Fig. 1d); therefore, the α6 helix of cFSP1 fails to accommodate Glu155 for selectively targeting 6-OH-FAD, in contrast to the observations in this work. Similarly, the S152A mutation in hFSP1 largely abrogates its 6-OH-FAD binding (Fig. 1b, g). The residue at the 355th position seems to always be Lys across poultry, reptiles, amphibians and mammals (Supplementary Fig. S5). In summary, the residues at equivalent positions to Ser152 and Lys355 of hFSP1 exhibit evolutionary variations from lower to higher organisms, particularly mammals.

Compared with that in hFSP1-6-OH-FAD, the isoalloxazine ring of 6-OH-FAD in the NAD(P)^+^ bound state undergoes a subtle shift of ~ 14.2° away from NAD(P)^+^ entry (Supplementary Fig. S6a, b) to contact the nicotinamide rings of NAD(P)H in an unusual mode. This permits the insertion of NAD(P)^+^ into the NAD(P)H-binding pocket. Strikingly, the nicotinamide ring of NAD(P)^+^ is oriented parallel to the *re*-face of the isoalloxazine ring and conjugates with Ring 1 (R1) of 6-OH-FAD (Supplementary Fig. S6g), with a 4.1 Å distance between the C4 group of the nicotinamide ring and the N5 group of the isoalloxazine (Supplementary Fig. S6c). This orientation and interaction are not observed in its orthologues, such as Ndi1, hAIF, and c.thNDH2, which utilize the R2 or R3 ring of FAD to engage the nicotinamide ring of NAD(P)^+^ (Supplementary Fig. S6d–f, h–j).

Moreover, the binding of NAD(P)^+^ also induces some conformational rearrangements in the 6-OH-FAD–binding domain of hFSP1. As a result, refiguration on the side chain of Met42 triggers the rotation of the pentose in 6-OH-FAD, enabling the 2-OH group on the pentose to form a hydrogen bond with Asn120 of hFSP1 (Supplementary Fig. S7a). In addition, the lateral movement on the side chain of Met294 further induces rotation of the isoalloxazine ring, in combination with the repositioning of Phe328, to promote electron transfer from the nicotinamide ring of NAD(P)^+^ onto the isoalloxazine ring of 6-OH-FAD (Supplementary Fig. S7b). Alternatively, conformational changes in the Thr243-Gly244-Ile245 motif can provide space for NAD(P)^+^ accommodation (Supplementary Fig. S7b). The side chain of Arg208, which is initially positioned within the previously reported phosphate-binding pocket in the absence of NAD(P)^+^^6^, tilts towards the NAD(P)H-binding domain to establish a hydrogen bond with the adenosine ribose of NAD(P)^+^.

Notably, the hydrogen bonding of Glu156 with 6-OH-FAD and NAD(P)^+^ drives them into proximity, allowing their direct contact (Supplementary Fig. S8a, b). Additionally, Lys355 stabilizes NAD(P)^+^ in a positively charged channel (Supplementary Fig. S9). To validate the structural observations, we introduced missense mutations into hFSP1. As a result, E156A and K355A compromise the oxidation activity for NADH and NADPH, indicating the importance of these two residues in NAD(P)H binding (Fig. 1i, j). In addition, we compared the structure of hFSP1-6-OH-FAD-NAD^+^ with that of hFSP1-6-OH-FAD-NADP^+^ and found only minor conformational differences (root-mean-square deviation of 0.104 Å), which is in line with our observations from the activity assay and a very recent claim by the Qi laboratory that hFSP1 is capable of oxidizing both NADH and NADPH at almost the same level^7^ (Fig. 1i, j and Supplementary Fig. S10a, b). In short, during this redox reaction process mediated by the 6-OH group, the conformational rearrangements in both hFSP1 and the isoalloxazine ring of 6-OH-FAD collectively promote distinct π–π packing interactions between the R1 ring of 6-OH-FAD and the nicotinamide ring of NAD(P)^+^. Both NADH and NADPH can serve as electron donors for 6-OH-FAD to initiate the redox process in hFSP1.

To achieve an advanced understanding of the molecular dynamics of the hFSP1 redox process, we systematically compared our hFSP1 structures with the present structures of FSP1. In contrast to our observations in this work and the structure of hFSP1-6-OH-FAD-NADP^+^ determined by Jia’s laboratory (PDB: 8JSC), the hFSP1-6-OH-NADH structure (PDB: 8WIK) published by the Qi’s laboratory shows that the nicotinamide ring of the reduced form of NADH is conjugated parallel to the R2 position of the isoalloxazine ring but not to R1 (Supplementary Fig. S10a, b). This may represent two distinct states during the reaction process catalysed by hFSP1: after and before oxidation, in which the nicotinamide ring of NAD(P)H can shuttle between the R1 and R2 rings along the plane. Furthermore, we utilized PyMOL to model the structure of cFSP1 complexed with CoQ_1_ (PDB: 7YTL) and observed that CoQ_1_ is located in the vicinity of the R3 ring (Supplementary Fig. S10c). In this scenario, we hypothesize that the nicotinamide ring of NAD(P)H initially conjugates with R2 to facilitate electron transfer and subsequently slides towards R1, providing enough space for efficient coenzyme Q accommodation. In addition, unlike the appropriate initial distance between the amino group at the 3rd position of the isoalloxazine ring and the carbonyl group of CoQ_1_ observed in the structure of cFSP1 bound to CoQ_1_, this corresponding distance in the model structure of hFSP1-6-OH-FAD-CoQ_1_ in the absence of NAD(P)^+^ is approximately 2.3 Å, which likely sterically blocks the contact between 6-OH-FAD and coenzyme Q. However, in the NAD(P)^+^ bound states, this distance expands to 2.9 Å owing to the rotation of the isoalloxazine ring and appears to be more conducive for coenzyme accommodation (Supplementary Fig. S10d). These findings collectively imply that hFSP1 possibly adopts a distinct electron transfer route to catalyse the reduction of coenzyme Q, in which NADH does not immediately dissociate from hFSP1 after transferring electrons to FAD but rather coexists with substrates and FAD in a noncanonical ternary complex.

To summarize, our structures first illustrate the molecular determinant of hFSP1 for preferential binding to 6-OH-FAD over FAD. The residue variations at equivalent positions to Ser152 and Lys355 of hFSP1 during evolution appear to coincide with selection for 6-OH-FAD and ferroptosis suppression in higher animals. Considering the correlation of reactive oxygen species with cellular ferroptosis, we speculate that the sequence variations in hFSP1 and the accompanying selection for 6-OH-FAD reflect the evolution of living cells at the molecular level to resist reactive oxygen species stress changes, not merely accidental cases. However, whether 6-OH-FAD is intrinsically associated with intracellular lipid peroxidation remains to be investigated. More importantly, hFSP1 presumably catalyses coenzyme Q reduction via an unrevealed mechanism. This work provides a comprehensive understanding of the mechanism of hFSP1 activation mediated by 6-OH-FAD and the dynamic electron transfer process in hFSP1 (Fig. 1k), guiding the future design of small-molecule inhibitors that target hFSP1 to prevent ferroptosis-associated disorders.

### Supplementary information

Supplementary Information

## Data Availability

All data needed to evaluate the conclusions in the paper are present in the paper and/or the Supplementary files. The reported crystal structures of hFSP1-6-OH-FAD, hFSP1-6-OH-FAD-NAD^+^, and hFSP1-6-OH-FAD-NADP^+^ have been deposited to the PDB under accession codes 8YOX, 8YO8, and 8YOQ, respectively.
