# Supplementary material for: Structural insight into 6-OH-FAD–dependent activation of hFSP1 for ferroptosis suppression
Source: Cell Discov. 2024 Aug 20;10:88. doi: 10.1038/s41421-024-00723-7 (PMC11333494; doi:10.1038/s41421-024-00723-7)
Supplement: Supplementary file 1 — Supplementary Information [file 41421_2024_723_MOESM1_ESM.pdf]

Supplementary Information

Structural insight into 6-OH-FAD Dependent Activation of hFSP1 for Ferroptosis Suppression

Hongying Lan[1], Yu Gao[1], Ting Hong[1], Zihan Chang[1], Zhengyang Zhao[1], Yanfeng Wang[1], Feng Wang[1*]

[1]Key Laboratory of Molecular Medicine and Biotherapy in the Ministry of Industry and Information Technology, Department of Biology, School of Life Sciences, Beijing Institute of Technology, Beijing, 100081, China.

[*]e-mail: Feng Wang, wfeng@bit.edu.cn

Materials and Methods

Supplementary Figures S1 – S10

Supplementary Table S1

Supplementary References

MATERIALS AND METHODS

Protein expression and purification

The recombinant hFSP1 gene sequence (residues 10-373, Uniprot entry: Q9BRQ8) was cloned into the pPGH vector with an N-terminal GST-HRV 3C tag and was subsequently transformed into *E. coli* BL21 (DE3) for overexpression. *E. coli* BL21 (DE3) cultures were grown in LB medium at 37°C. When the OD_{600} reached 0.8, the culture was induced at 25°C for 14-16 hours with 1 mM isopropyl β-D-thiogalactopyranoside. The bacterial cells were harvested by centrifugation at 4000 rpm (4°C) for 20 minutes, followed by next steps: centrifugation, resuspension, and sonication in buffer A (50 mM K_3PO_4, pH 8.0, 300 mM KCl, 0.1% (v/v) Triton X-100, 1 mM dithiothreitol). Subsequently, ultrafast centrifugation (16000 rpm, 4°C, 50 min) was performed to remove cell fragments. The resulting supernatant was then loaded onto a GST affinity column pre-equilibrated with buffer A. Non-specifically bound proteins were then eluted using buffer A. The GST tag was cleaved from hFSP1 at room temperature using HRV 3C protease in the presence of buffer A for 4 hours. Subsequently, the resin was eluted with buffer B (50 mM K_3PO_4, pH 8.0, 300 mM KCl, 1 mM dithiothreitol), and the collected protein was concentrated to 40 mg/mL using a 30 kDa concentration tube. The final purification step was conducted on a Superdex 200 Increase 10/300 GL column (GE Healthcare, Sweden), pre-equilibrated with a buffer (25 mM Tris-HCl, pH 8.0, 300 mM NaCl, 0.012% (w/v) n-dodecyl β-D-maltoside). Protein located at the peak apex was collected for subsequent biochemical experiments and protein crystallization. The hFSP1 variant (including D41A, N49A, S152A, E156A, E156S, E156D, E156Q, S175A, Q176A, R208A, N248A, D285A, F328A, and K355A) plasmids were generated by PCR using the Mut Express II Fast Mutagenesis Kit V2 (Vazyme, China), and all the proteins were overexpressed and purified by the same method. Protein concentration was determined by SDS-PAGE combined with absorbance measurement at 280 nm by spectrophotometry.

Crystallization, data collection, and structure determination

The crystallization trials were conducted using the hanging-drop vapor-diffusion methods at 18°C, with all hFSP1 proteins at the concentration of 15 mg/mL. The crystals with the highest resolution were achieved after two weeks to one month in the reservoir solution containing 100 mM NaCl, 120 mM Li_2SO_4, 100 mM Sodium HEPES (pH 7.7) and 16% (w/v) PEG 6000, and then flash-frozen in liquid nitrogen with a cryoprotectant consisting of the reservoir solution supplemented with 20% (v/v) ethylene glycol. In the crystallization trials for hFSP1 bound to $NAD(P)^+$, we add $NAD(P)^+$ (MedChemExpress) to hFSP1 with a final concentration of 1.2 mM, and incubate for 40 minutes prior to initiating experiment.

All data collection was conducted at the Shanghai Synchrotron Radiation Facility beamlines BL19U1 or BL02U1. The collected data were integrated and scaled using the HKL3000 software packages. Subsequent data processing was carried out utilizing the CCP4 suite[1,2]. The structure for hFSP1-6-OH-FAD was determined by molecular displacement using the structure predicted by AlphaFold as an initial model with

PHENIX[3,4]. An iterative optimization and refinement process was employed with PHENIX and COOT to obtain the final structural model. Molecular substitution was performed for hFSP1-6-OH-FAD-NAD$^+$ and hFSP1-6-OH-FAD-NADP$^+$ using hFSP1-6-OH-FAD as a reference model. Detailed data collection and structure refinement statistics can be found in Supplementary Table S1. The analysis and visualization of the structure were performed using PyMOL (https://pymol.org/2).

Enzyme assays for hFSP1

Enzyme activity hFSP1 (NAD(P)H oxidation activity) was measured by detecting the decrease of NAD(P)H absorbance at 340 nm using the multimode plate reader (Infinite F Nano+, Tacan). The assay was performed at a temperature of 37℃ in a buffer system, which contains 50 mM Tris-HCl (pH 7.5), 300 mM NaCl, and 0.012% (w/v) n-dodecyl β-D-maltoside. The final reaction mixture in a total volume of 100 μL comprises 0.25 μM hFSP1, 500 μM NAD(P)H, and 250 μM menadione. The absorbance at 340 nm was monitored at 37℃ for 25 minutes continuously, and recorded once every 10 seconds. Each assay was repeated for at least three times.

Ultraviolet–visible spectroscopy assays

Ultraviolet–visible spectroscopy assays were conducted at room temperature using the spectrophotometer (UV-3900, HITACHI). The detected ultraviolet wavelength varies from 300 to 800 nm, and protein concentrations in the range of approximately 2.5-4.5 mg/mL were utilized.

Extraction and identification of cofactors in hFSP1

Purified proteins are processed through a desalination procedure (dilute with ddH$_2$O and concentrate), followed by precipitation with 5% trichloroacetic acid. After that, the sample was centrifuged with a microcentrifuge at 15000 rpm (4°C) for 15 minutes. This precipitation step was repeated several times until all the protein was completely removed. The final supernatant solution was placed in a -80°C freezer for 3-5 hours and subsequently processed through freeze-drying for a period of 14-16 hours to obtain the cofactor.

The extracted cofactors were dissolved in ddH$_2$O, and identified using MALDI-TOF mass spectrometry (Autoflex Bruker). α-Cyano-4-hydroxycinnamic acid (4-HCCA) serves as the matrix for MALDI-TOF mass spectrometry detection, mixed with the cofactor solution in a 1:1 volume ratio. Subsequently, 2 μL of the mixture is applied to the MALDI plate, and subject to natural evaporation for approximately 5 minutes. The analysis was conducted in linear negative ion mode.

Supplementary Figures

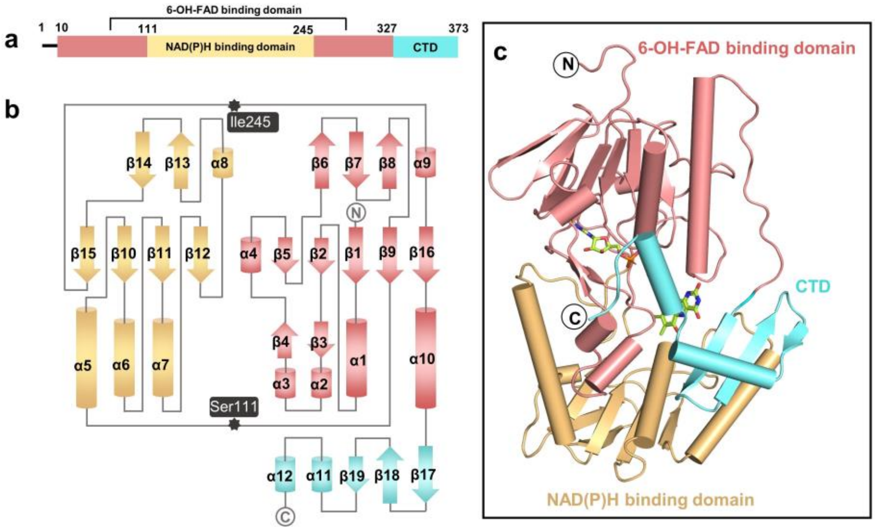

Supplementary Fig. S1. Overall structures of hFSP1. a, Schematic representation of hFSP1. The region spanning residues 1-10, that appears structurally disorder, harbors myristoylation site and directs hFSP1 for membrane anchor. The 6-OH-FAD-binding domain (residues 10-111 and 246-327) is shown in pink. The NAD(P)H-binding domain (residues 112-245) is shown in yellow. The C-terminal membrane-anchoring domain (CTD, residues 328-373) is shown in cyan. **b**, Topology diagram of hFSP1. The pink arrows, cyan cylinders, and black lines represent α-helices, β-strands, and loops, respectively. **c**, Cartoon representation of hFSP1. The coloration of the hFSP1 domains aligns with that depicted in the (a).

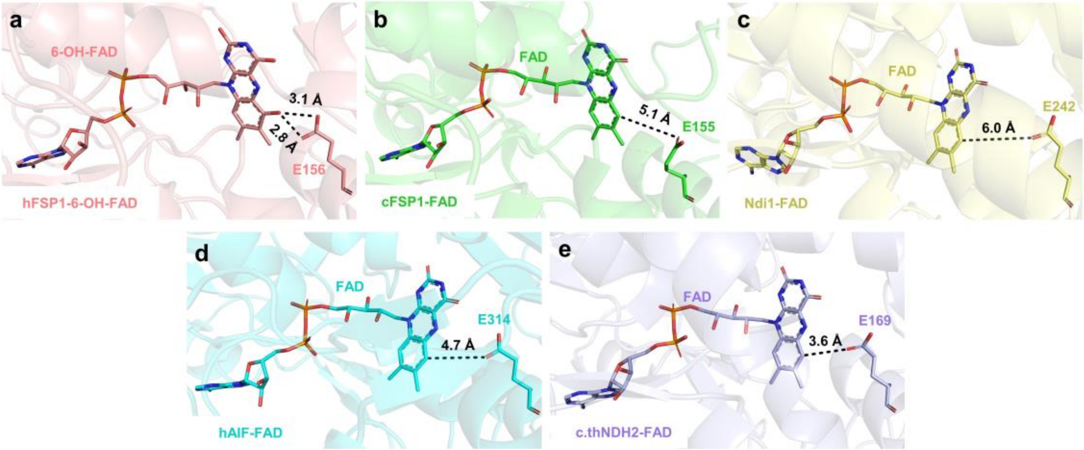

Supplementary Fig. S2. The distinctive interaction involving E156 and 6-OH-FAD in hFSP1. **a**, The distance between the carbonyl and hydroxyl groups of E156 and the hydroxyl group on the 6th carbon of 6-OH-FAD's isoalloxazine ring in hFSP1-6-OH-FAD is 2.8 Å and 3.1 Å, respectively. **b**, The distance between the 6th carbon of FAD's isoalloxazine ring and the carboxyl group of E155 in cFSP1-FAD (PDB: 7XPI) is 5.1 Å. **c**, The distance between the 6th carbon of FAD's isoalloxazine ring and the carboxyl group of E242 in Ndi1-FAD (PDB: 4G6G) is 5.1 Å. **d**, The distance between the 6th carbon of FAD's isoalloxazine ring and the carboxyl group of E314 in hAIF-FAD (PDB: 1M6I) is 4.7 Å. **e**, The distance between the 6th carbon of FAD's isoalloxazine ring and the carboxyl group of E169 in c.thNDH2-FAD (PDB: 4NWZ) is 3.6 Å.

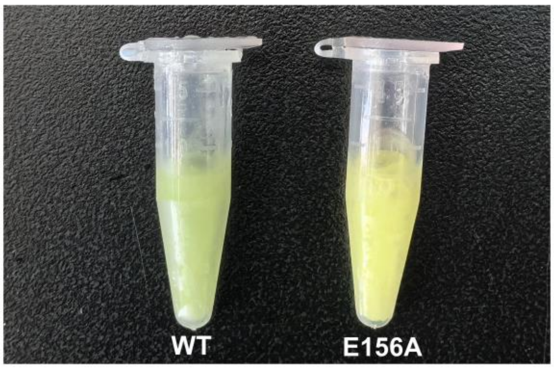

Supplementary Fig. S3. The colour difference between hFSP1 (left) and E156A (right).

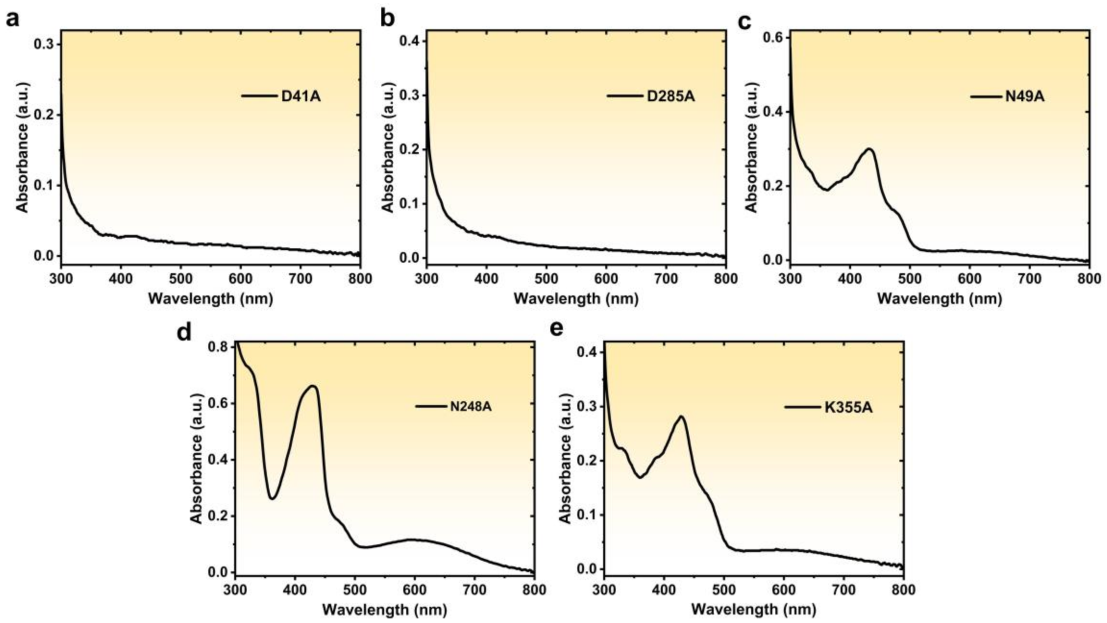

Supplementary Fig. S4. The Ultraviolet–visible spectroscopy measurement of five hFSP1 variants. a, D41A. **b**, D285A. **c**, N49A. **d**, N248A. **e**, K355A.

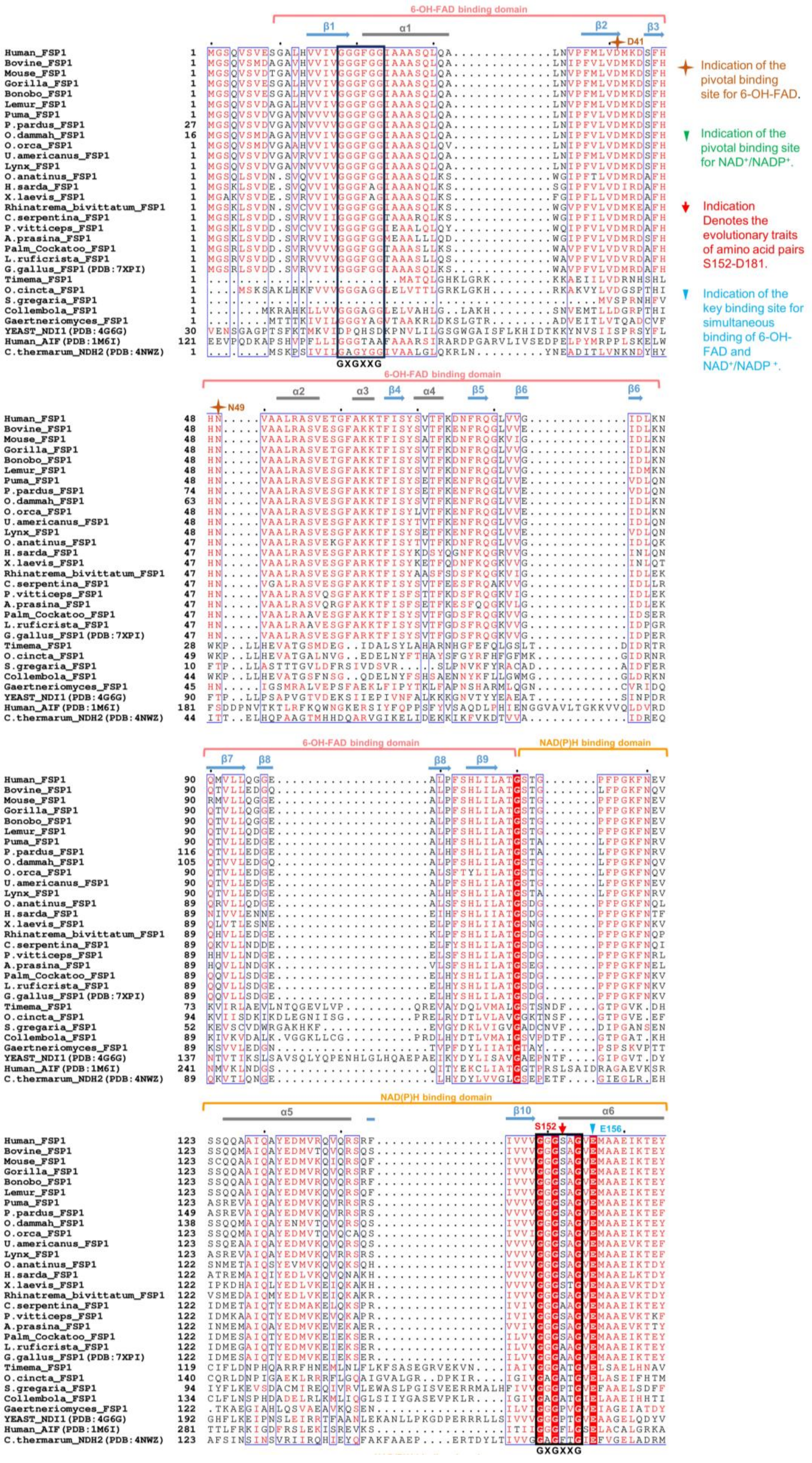

6-OH-FAD binding domain
NAD(P)H binding domain
GXGXXG
D41
N49
S152
E156
Indication of the pivotal binding site for 6-OH-FAD.
Indication of the pivotal binding site for NAD+/NADP+.
Indication Denotes the evolutionary traits of amino acid pairs S152-D181.
Indication of the key binding site for simultaneous binding of 6-OH-FAD and NAD+/NADP+.

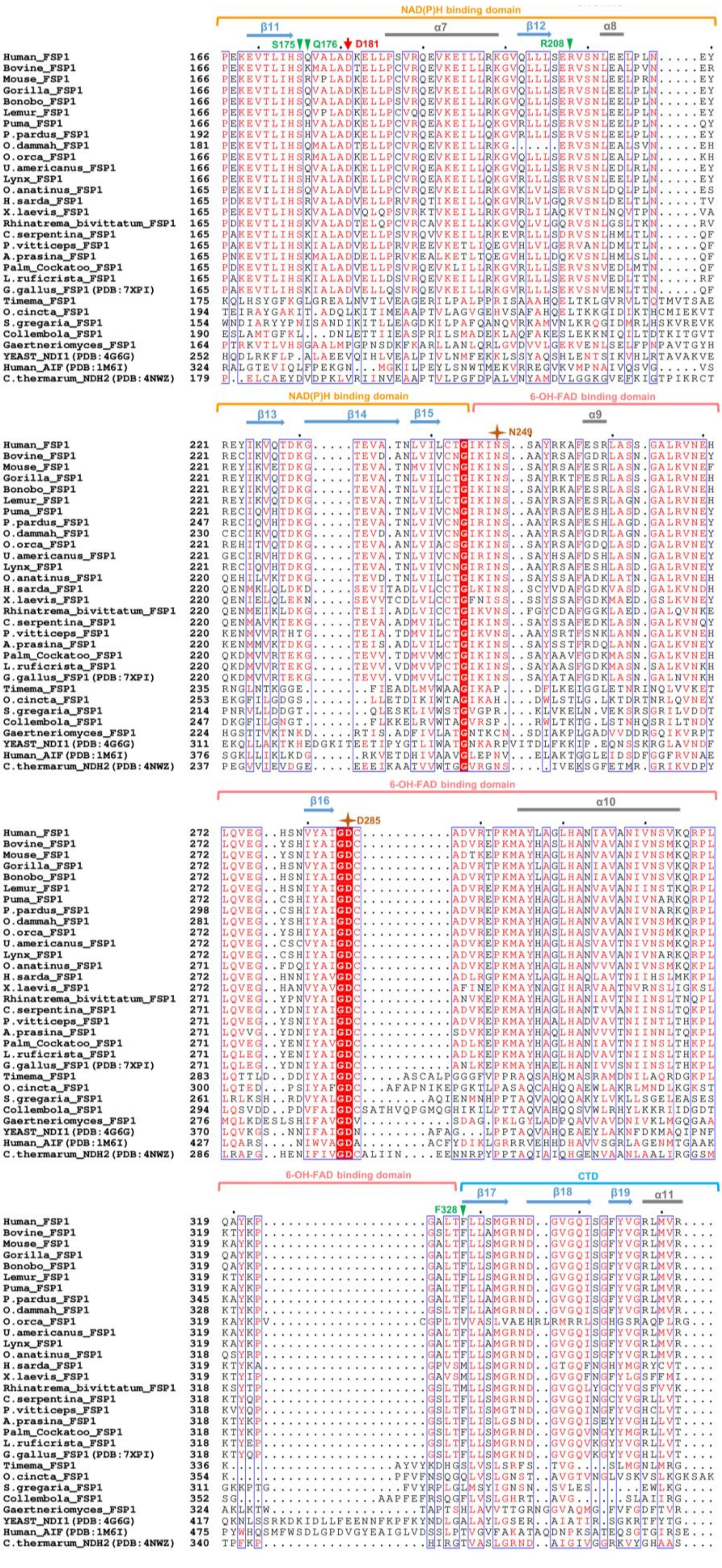

NAD(P)H binding domain
β11
α7
β12
α8
S175
Q176
D181
R208
β13
β14
β15
6-OH-FAD binding domain
α9
N249
β16
D285
α10
CTD
F328
β17
β18
β19
α11

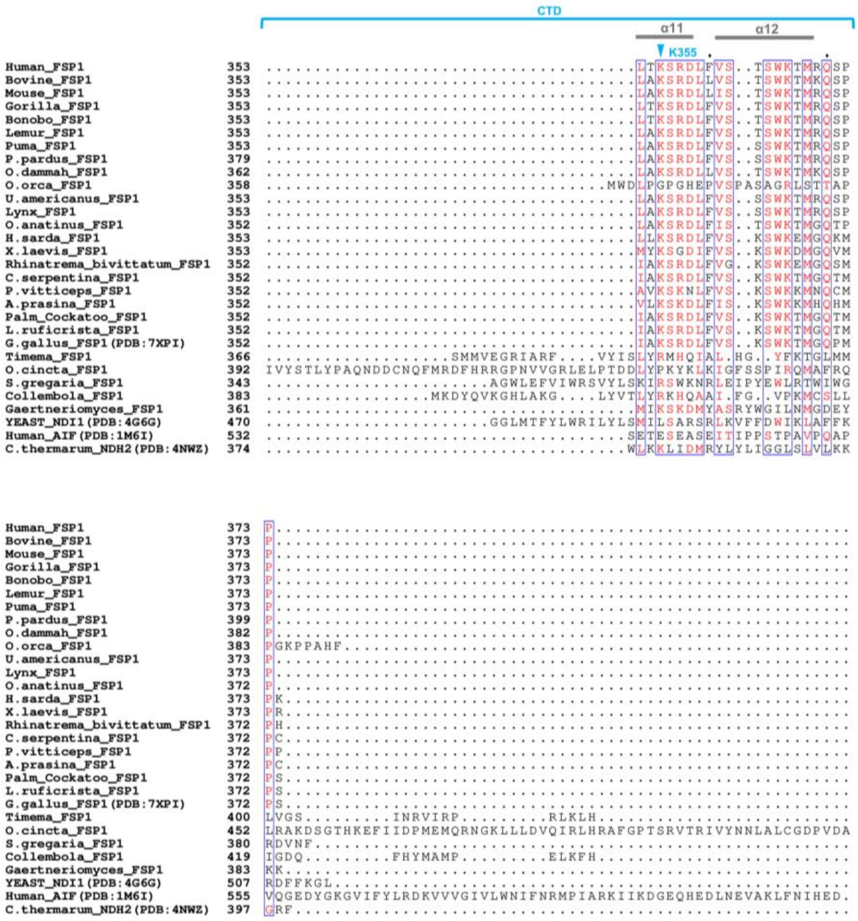

Supplementary Fig. S5. Sequence alignment of FSP1 with its homologous proteins from various species, including Ndi1, hAIF, and c.thNDH2. The structural domains of hFSP1 are shown with curly braces, above the sequences. The 6-OH-FAD (residues 10-111 and 245-327) and NAD(P)H (residues 112-245) binding domain are indicated by pink and yellow curly braces, respectively. The C-terminal membrane anchoring domain (CTD, residues 328-373) is indicated by cyan curly braces. The observed secondary structural elements of hFSP1 are annotated. The α-helix regions and β-sheets are indicated by blue arrows and gray bars, respectively. The GXGXXG motifs in the Rosmann folds are highlighted in black boxes. The brown-yellow stars signify the crucial amino acids within the 6-OH-FAD binding pocket, the green triangles represent the vital amino acids in the NAD(P)H binding pocket, the blue triangles represent the amino acids capable of binding to both 6-OH-FAD and NAD(P)H (E156 and K355), while the red arrows represent S152 and D181. P.pardus: Panthera pardus, O.dammah: Oryx dammah, O.orca: Orcinus orca, U.americanus: Ursus americanus, O.anatinus: Ornithorhynchus anatinus, H.sarda: Hyla sarda, X.laevis: Xenopus laevis, C.serpentina: Chelydra serpentina, P.vitticeps: Pogona vitticeps, A.prasine: Ahaetulla prasina, L.ruficrista: Lophotis ruficrista, G.gallus: Gallus.gallus, O.cincta: Orchesella cincta, S.gregaria: Schistocerca gregaria, C.thermarum_NDH2: *Caldalkalibacillus thermarum* NDH2.

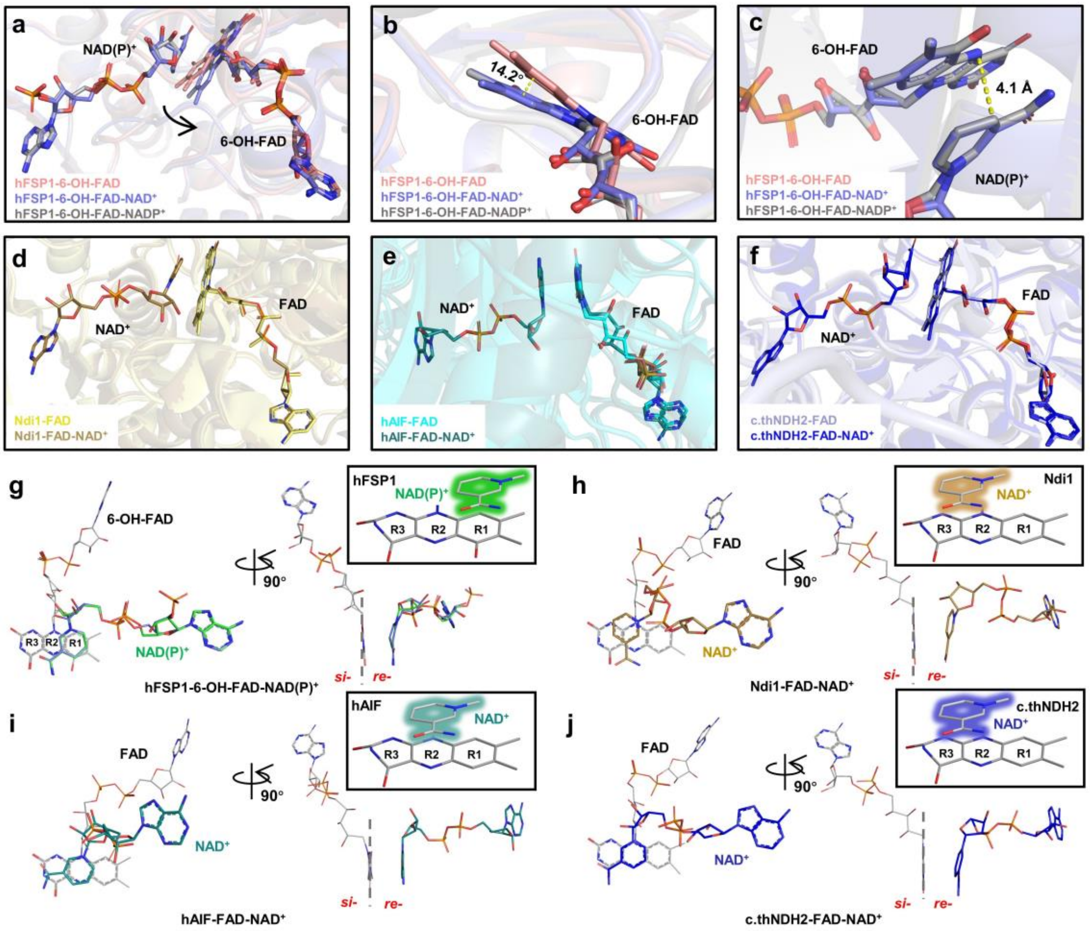

Supplementary Fig. S6. The distinct interaction mode of 6-OH-FAD and NAD(P)$^+$ in hFSP1. a, Structural overlay of 6-OH-FAD in hFSP1-6-OH-FAD with that in hFSP1-6-OH-FAD-NAD(P)$^+$. The structures of hFSP1-6-OH-FAD, hFSP1-6-OH-FAD-NAD$^+$ and hFSP1-6-OH-FAD-NADP$^+$ are depicted by pink, purple, and gray, respectively. The black arrow indicates the deviation direction of the isoalloxazine ring in 6-OH-FAD after NAD(P)$^+$ binding. **b**, NAD(P)$^+$ binding to hFSP1 results in a 14.2° deviation of the isoalloxazine ring in 6-OH-FAD. **c**, The distance between the C4 of NAD(P)$^+$ nicotinamide ring and the N5 of 6-OH-FAD isoalloxazine ring is 4.1 Å. **d**, Structural overlay of the FAD in Ndi1-FAD (in light yellow, PDB: 4G6G) with that in Ndi1-FAD-NAD$^+$ (in brown-yellow, PDB: 4G6H). **e**, Structural overlay of FAD in hAIF-FAD (in cyan, PDB: 4BV6) with that in hAIF-FAD-NAD$^+$ (in teal, PDB: 4BUR). **f**, Structural overlay of FAD in c.thNDH2-FAD (in light purple, PDB: 4NWZ) with that of c.thNDH2-FAD-NAD$^+$ (in deep purple, PDB: 5KMS). **g-j**,The distinct interaction mode between the nicotinamide ring of NAD(P)$^+$ and isoalloxazine ring of FAD (including 6-OH-FAD) bound to distinct NDH-2: hFSP1 (**g**), Ndi1 (**h**), hAIF (**i**), and c.thNDH2 (**j**). FAD (including 6-OH-FAD) is depicted in a gray line. hFSP1-6-OH-FAD-NAD$^+$ is displayed by purple, hFSP1-6-OH-FAD-NADP$^+$ by green, Ndi1-FAD-NAD$^+$ by brown-yellow, hAIF-FAD-NAD$^+$ by teal, and c.thNDH2-FAD-NAD$^+$ by deep purple. "*si*-" and "*re*-" denote the *si*- or *re*- face of the isoalloxazine ring. The illustration in the upper right corner represents a simplified diagram for the interaction mode between the nicotinamide ring and the isoalloxazine ring.

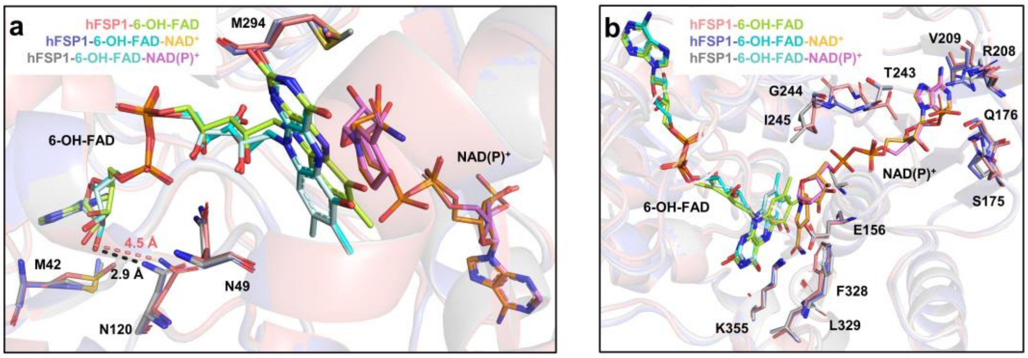

Supplementary Fig. S7. The conformational changes of hFSP1 binding to NAD (P)$^{+}$. a, Structural superposition of the 6-OH-FAD binding pocket (M42, N49, N120, and M294) in hFSP1-6-OH-FAD, hFSP1-6-OH-FAD-NAD^{+}, and hFSP1-6-OH-FAD-NADP^{+}. In hFSP1-6-OH-FAD, the distance between 6-OH-FAD and the side chain of N120 is 4.5 Å and indicated by the pink dashed line. The distance in the hFSP1-6-OH-FAD-NAD(P)$^{+}$ is 2.9 Å and depicted by the black dashed line. In hFSP1-6-OH-FAD, 6-OH-FAD is shown in lemon green, and the amino acids in the NAD(P)H binding pocket in pink. In hFSP1-6-OH-FAD-NAD^{+}, 6-OH-FAD and NAD^{+} are shown in cyan and orange, while the amino acids in the NAD(P)H binding pocket in purple. In the hFSP1-6-OH-FAD-NADP^{+} complex, 6-OH-FAD and NADP^{+} are indicated by pale cyan and magenta, and the corresponding amino acids in the NAD(P)H binding pocket by gray. **b**, Structural superposition of the NAD(P)$^{+}$ binding pocket (E156, S175, Q176, R208, V209, T243, G244, I245, F328, L329, and K355) in the hFSP1-6-OH-FAD, hFSP1-6-OH-FAD-NAD^{+}, and hFSP1-6-OH-FAD-NADP^{+}. Colour codes are the same as in the scheme (a).

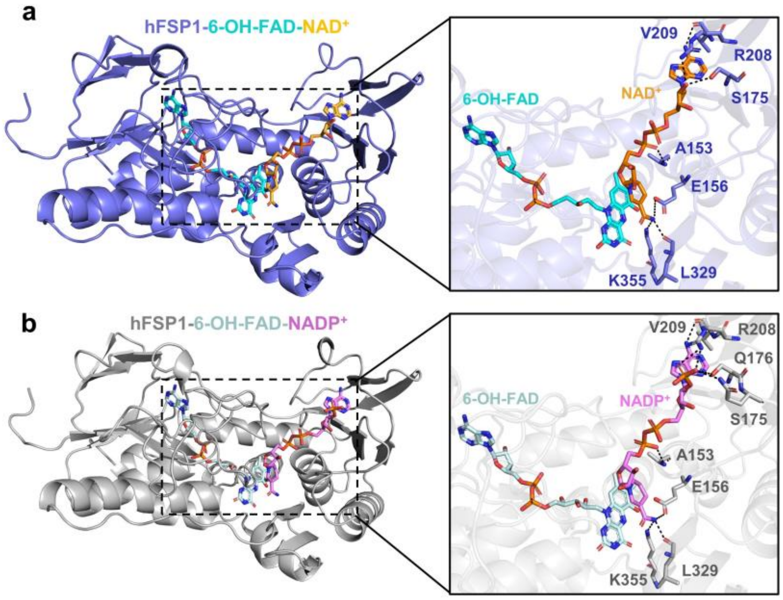

Supplementary Fig. S8. The binding sites of NAD$^+$ and NADP$^+$ in hFSP1. a, Overall structures of hFSP1-6-OH-FAD-NAD$^+$ (left), and the NAD$^+$ binding pocket is zoomed in (right). hFSP1 is shown in purple ribbon, the molecules 6-OH-FAD and NAD$^+$ are shown as the cyan and orange sticks, respectively. The hydrogen bond interactions are depicted by black dashed lines. **b**, Overall structure of hFSP1-6-OH-FAD-NADP$^+$ (left) and the NADP$^+$ binding pocket is zoomed in (right). hFSP1 is shown in a gray cartoon, with 6-OH-FAD as pale cyan sticks and NADP$^+$ as magenta sticks. The black dashed lines represent hydrogen bonds.

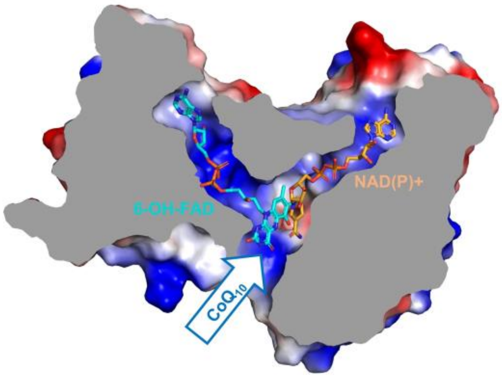

Supplementary Fig. S9. The substrate-binding channel of hFSP1. The electrostatic surface representation for the binding channels in hFSP1 to 6-OH-FAD, NAD(P)H, and CoQ_{10}. 6-OH-FAD and $NAD(P)^+$ are indicated by cyan and orange, while CoQ_{10} is symbolized using a blue arrow.

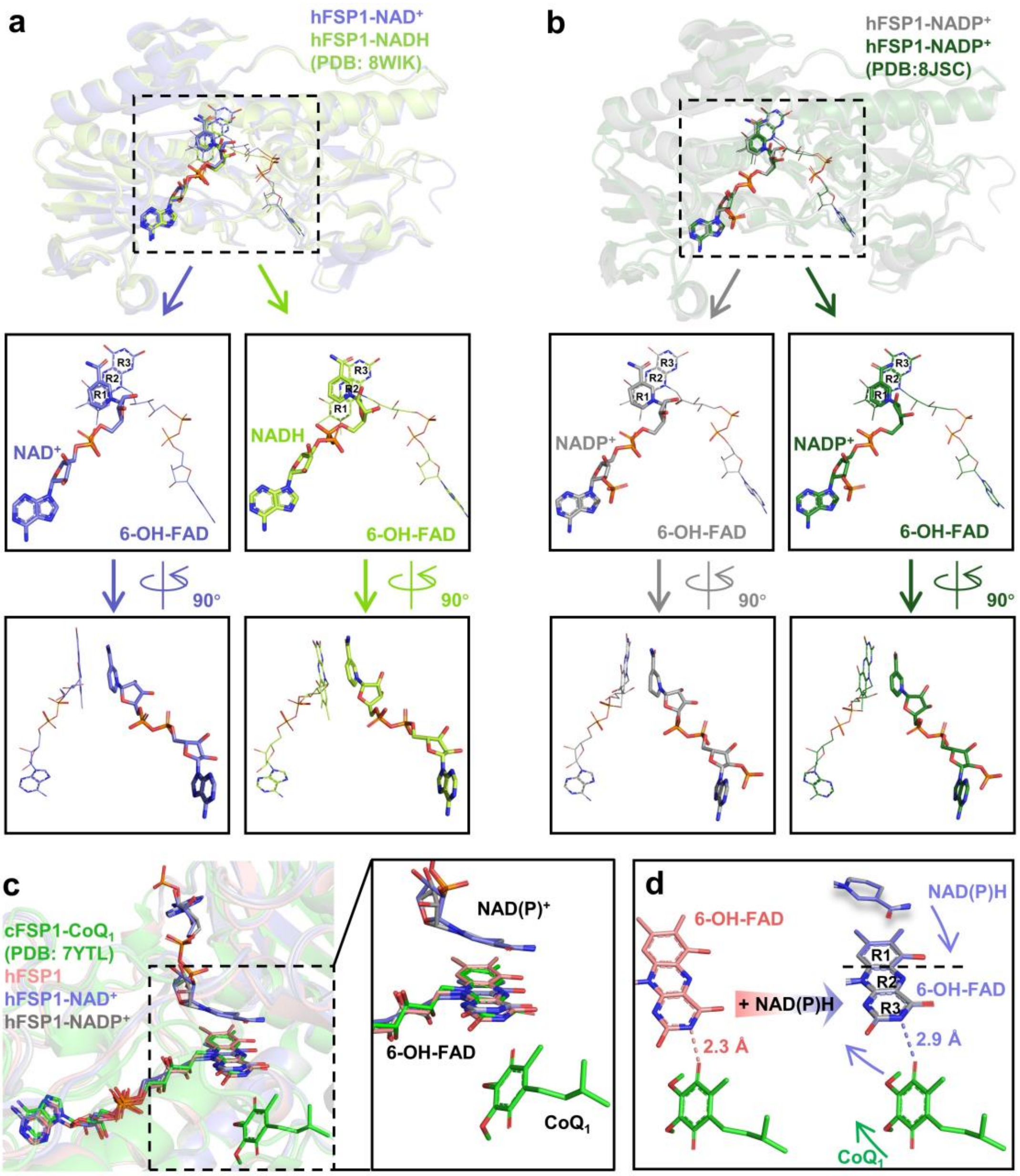

Supplementary Fig. S10. The redox mechanism of hFSP1. a, Structural superposition of hFSP1-6-OH-FAD-NAD$^+$ (hFSP1-NAD$^+$, in purple) and hFSP1-6-OH-FAD-NADH (PDB: 8WIK, hFSP1-NADH, in lemon green). **b**, Structural superposition of hFSP1-6-OH-FAD-NADP$^+$ (hFSP1-NADP$^+$, in gray) and hFSP1-6-OH-FAD-NADP$^+$ (PDB: 8JSC, hFSP1-NADP$^+$, in dark green). **c**, The modeling of cFSP1-FAD-CoQ$_1$ structure (cFSP1-CoQ$_1$, PDB: 7YTL, in green) into hFSP1-6-OH-FAD (hFSP1, in pink), hFSP1-6-OH-FAD-NAD$^+$ (hFSP1-NAD$^+$, in purple), and hFSP1-6-OH-FAD-NADP$^+$ (hFSP1-NADP$^+$, in gray) utilizing PyMOL. **d**, The hypothesis of the redox transfer process in hFSP1. Here, the spatial coordinate of NAD(P)$^+$ is utilized to denote that of NAD(P)H. The 6-OH-FAD of hFSP1-6-OH-FAD is shown as pink, the 6-OH-FAD and NADH of hFSP1-6-OH-FAD-NAD$^+$ are shown as purple, and the 6-OH-FAD and NADPH of hFSP1-6-OH-FAD-NADP$^+$ are shown as gray. When the NAD(P)H binds, the isoalloxazine ring in 6-OH-FAD twists around the carbon chain between Ring1 (R1) and Ring2 (R2) to accommodate CoQ$_1$ entry, which

is indicated by purple arrows. The rotation axis is indicated by the black dashed line. The green arrow denotes the trajectory for entry of CoQ_1 into the active site pocket. In the hFSP1-6-OH-FAD, the distance from the N4 of the 6-OH-FAD's isoalloxazine ring to the carbonyl of CoQ_1 is 2.3 Å, while this distance in hFSP1-6-OH-FAD-NAD(P)$^+$ extends to 2.9 Å.

Supplementary Table S1. Data collection and refinement statistics.

	hFSP1-6-OH-FAD (PDB: 8YOX)	hFSP1-6-OH-FAD-NAD$^+$ (PDB: 8YO8)	hFSP1-6-OH-FAD-NADP$^+$ (PDB: 8YOQ)
**Data collection**			
Space group	P 32 2 1	P 32 2 1	P 32 2 1
Cell dimensions			
a, *b*, *c* (Å)	60.36 60.36 194.38	60.162 60.162 195.256	60.302 60.302 194.721
α, β, γ (°)	90 90 120	90 90 120	90 90 120
Resolution (Å)	27.54-2.48 (2.569-2.48)	32.54-2.00 (2.07-2.00)	32.45-1.88 (1.947-1.88)
R_{sym} or R_{merge}	0.092(0.393)	0.129(1.774)	0.293(6.594)
$I / \sigma I$	19.3(2.9)	25.68(2.29)	33.27(2.82）
Completeness (%)	98.5	84	94.3
Redundancy	14.9	19.2	18.9
**Refinement**			
Resolution (Å)	27.54-2.48	32.54-2.00	32.45-1.88
No. reflections	15119	28766	40126
R_{work} / R_{free}	0.184/0.259	0.196/0.236	0.188/0.233
No. atoms			
Protein	2747	2766	2744
Ligand/ion	54	103	107
Water	24	129	290
B-factors			
Protein	30.74	31.22	21.86
Ligand/ion	31.22	27.81	19.13
Water	35.16	32.52	30.75
R.m.s. deviations			
Bond lengths (Å)	0.003	0.015	0.015
Bond angles (°)	0.63	1.12	1.35
Ramachandran favored (%)	96.44	97.81	96.44
Ramachandran allowed (%)	3.56	2.19	3.56
Ramachandran outliers (%)	0.00	0.00	0.00
Rotamer outliers (%)	2.46	1.05	0.00

SUPPLEMENTARY REFERENCES

1 Minor, W., Cymborowski, M., Otwinowski, Z. & Chruszcz, M. HKL-3000: the integration of data reduction and structure solution - from diffraction images to an initial model in minutes. *Acta Crystallographica Section D* **62**, 859-866 (2006).

2 Winn, M. D. *et al.* Overview of the CCP4 suite and current developments. *Acta Crystallographica Section D* **67**, 235-242 (2011).

3 Jumper, J. *et al.* Highly accurate protein structure prediction with AlphaFold. *Nature* **596**, 583-589 (2021).

4 Varadi, M. *et al.* AlphaFold Protein Structure Database: massively expanding the structural coverage of protein-sequence space with high-accuracy models. *Nucleic Acids Res.* **50**, D439-D444 (2022).
